# Sol-gel Autocombustion Synthesis of Nanocrystalline High-entropy Alloys

**DOI:** 10.1038/s41598-017-03644-6

**Published:** 2017-06-13

**Authors:** Bo Niu, Fan Zhang, Hang Ping, Na Li, Jieyang Zhou, Liwen Lei, Jingjing Xie, Jinyong Zhang, Weimin Wang, Zhengyi Fu

**Affiliations:** State Key Laboratory of Advanced Technology for Materials Synthesis and Processing, Wuhan University of Technology, Wuhan, 430070 China

## Abstract

A reduction in the particle size is expected to improve the properties and increase the application potential of high-entropy alloys. Therefore, in this study, a novel sol–gel autocombustion technique was first used to synthesize high-entropy alloys. The average grain size of the prepared nanocrystalline CoCrCuNiAl high-entropy alloys showed was 14 nm with an excellent and uniform dispersion, exhibiting a distinct magnetic behavior similar to the superparamagnetic behavior. We show that the metal nitrates first form (Co,Cu,Mg,Ni,Zn)O high-entropy oxides, and then *in situ* reduce to CoCrCuNiAl high-entropy alloys by the reducing gases, and the chelation between citric acid and the metal ions and the *in situ* chemical reactions are the dominant reaction mechanisms. We demonstrate that the sol–gel autocombustion process is an efficient way to synthesize solid solution alloys eluding the restriction of a high mixing entropy.

## Introduction

Over the past years, high-entropy alloys (HEAs) have received more and more attention owing to their unique compositions, microstructures and adjustable properties^1^. In contrast to traditional alloys based on only one or two principal elements, HEAs were originally defined as multicomponent alloys composed of five or more principal elements in an equal or near-equal ratio^2, 3^. The resulting high mixing entropy was demonstrated to facilitate the formation of simple solid solutions rather than complex phases^4^. HEAs typically exhibit excellent physicochemical properties such as special electrical and magnetic properties^5, 6^, a high strength as well as promising resistances to wear, oxidation and corrosion^7–9^, making them promising candidates for future engineering applications.

A variety of synthesis techniques have aided the development of HEAs such as arc melting^10^, laser cladding^11^ and thin-film sputtering^12^. However, these fabrication routes are sometimes unsuitable for industrial manufacturing, because they might prove uneconomic or impose restrictions on the shape (bulk or film) or the size of the final product. Metallic nanoparticles have attracted significant attention from both the academia and the industry because of their unique physicochemical properties (e.g., their magnetic, catalytic and biomedical properties) arising from their much smaller size compared to their bulk counterparts^13, 14^. To date, HEA nanoparticles are generally synthesized via mechanical alloying (MA). However, when using this method, the grains are usually larger than 30 nm and the nanograins tend to agglomerate and form particles larger than 3 μm^15, 16^. A size reduction of the particles is expected to improve the properties and increase the application potential of HEAs. This, however, requires the development of a new synthesis method to prepare nanocrystalline HEAs.

Sol–gel autocombustion is a rapid and economical synthesis technique for the fabrication of particulate products and has been widely used for the synthesis of a variety of metal and alloy nanoparticles, forming nano-sized, homogeneous, and highly reactive powders through mixing different elements at the atomic level^17^. For instance, Yang *et al*. successfully synthesized pure Co, Ni, Cu, Ag, and Bi metals with a grain size of several nanometers via sol–gel autocombustion^18^. Pure metal atoms can be used as building blocks in the construction of different types of alloys. In previous studies, CoNi, NiFe, and even immiscible NiAg nanoalloys have been successfully prepared by the sol–gel autocombustion method^19–21^, indicating a great potential for the synthesis of nanocrystalline HEAs.

In this study, the feasibility of this approach was demonstrated using the CoCrCuNiAl HEA as an example. As one of the first identified HEAs^22^, CoCrCuNiAl contains five metals with different crystal structures (Cu, Ni and Al crystallize in the face-centered cubic (FCC) structure, Cr in the body-centered cubic (BCC), and Co in the hexagonal close-packed (HCP) structure) and consists of simple solid solutions of the FCC and BCC structure. In this study, CoCrCuNiAl HEA was synthesized via sol-gel autocombustion, and the effect of the fuel-oxidant ratio on the combustion process and the final products was investigated. In addition, the microstructure and the magnetic properties of the prepared CoCrCuNiAl HEA were studied.

## Results and Discussion

**Figure 1:**
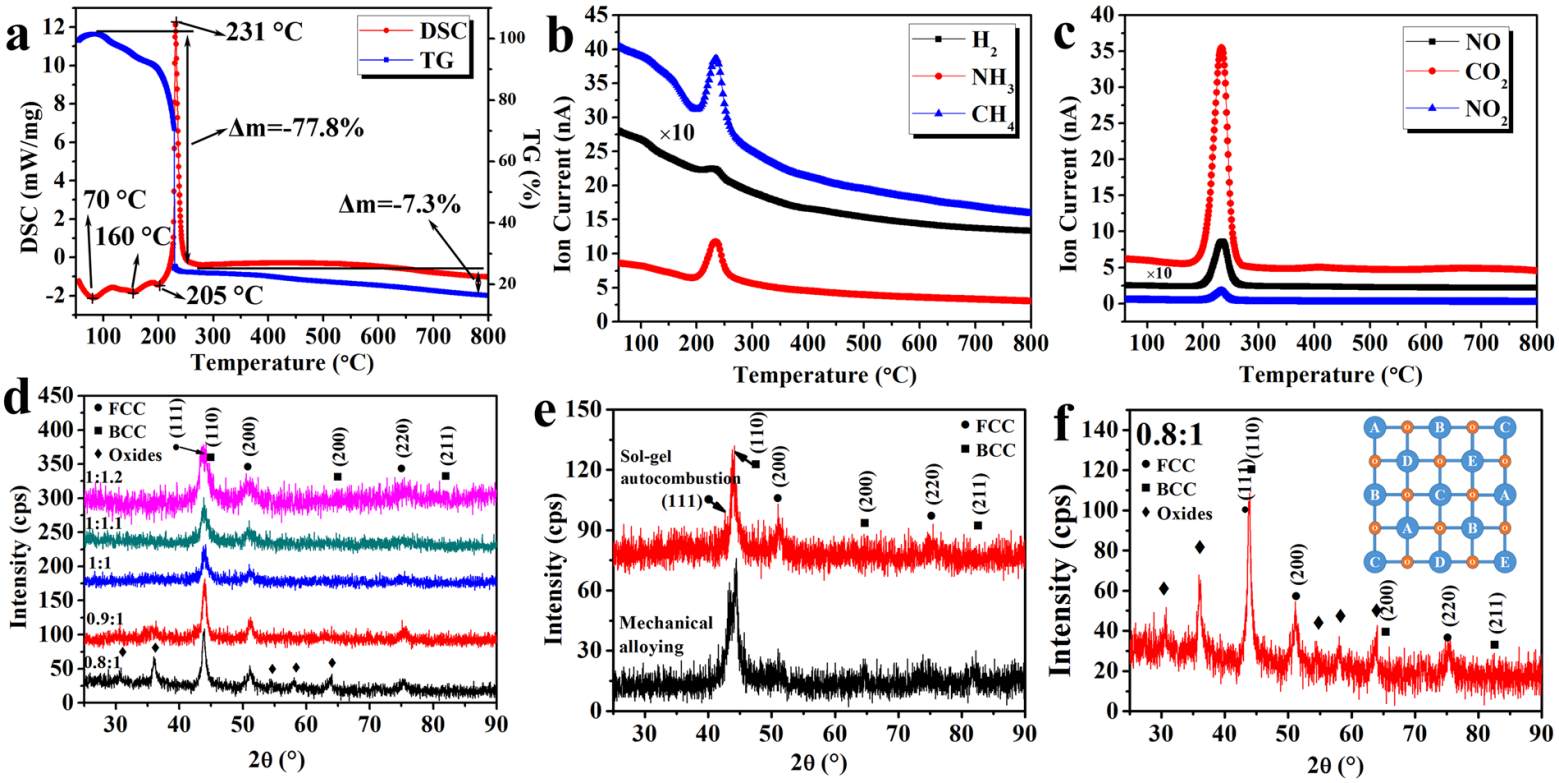
(**a**–**c**) TG–DSC–MA results obtained for the dried gel prepared from the sol with a fuel–oxidant ratio of 1:1; (**d**) XRD patterns revealing the crystal structures of the combustion products of the dried gels for different fuel-oxidant ratios; (**e**) comparison of XRD patterns of CoCrCuNiAl HEAs synthesized by sol–gel autocombustion and MA; (**f**) XRD patterns of the combustion product of the gels with a fuel–oxidant ratio of 0.8:1 and the schematic illustration of HEOs.

The sol–gel autocombustion technique is primarily used to synthesize nanosized oxides via an exothermic reaction between oxidants (typically metal nitrates) and fuel (such as organic amines, urea and acids)^23^. In this study, a nitrate–citric acid system was used, with the nitrate ions acting as the oxidants and the citric acid as the fuel. To further understand the reaction process, a thermogravimetric–differential scanning calorimetry–mass spectrometry (TG–DSC–MS) analysis was performed to analyze the combustion reaction, and the gas composition of the dried gel with a fuel-oxidant ratio of 1:1. As shown in Fig. 1, the endothermic peaks appeared at ∼70 and 130 °C. They correspond to the evaporation of the free and the bound water in the dried gels, respectively. Another endothermic peak was observed at a temperature of 205 °C and may be ascribed to the decomposition of NH_4_NO_3_ formed during the adjustment of the pH value. A strong exothermal peak associated with a sharp decrease in the weight was observed at 231 °C, related to the combustion reaction. No additional peaks but only a small reduction in weight were observed after the combustion process, indicating the formation of a stable phase. As shown in Fig. 1b and c, during the combustion process, gases such as CO_2_, NO_2_, and NO are released, and the metal oxides will be formed simultaneously through the decomposition of the nitrates. Meanwhile, reduction gases such as H_2_, CH_4_, and NH_3_ are released as well and play an important role in the reduction of the metal oxides according to a previous study^24^. The combustion reaction can be simply described as follows (where *M* denotes the metal atom/ions and *n* is the metal valence):

1 $$2N{H}_{4}N{O}_{3}\to 2N{H}_{3}\uparrow +N{O}_{2}\uparrow +NO\uparrow +{H}_{2}O\uparrow +{O}_{2}\uparrow $$

2 $${C}_{6}{H}_{8}{O}_{7}+{H}_{2}O\to 4C{O}_{2}\uparrow +2C{H}_{4}\uparrow +{H}_{2}\uparrow \,$$

3 $${\rm{M}}{({{\rm{NO}}}_{3})}_{n}+{C}_{6}{H}_{8}{O}_{7}+(\frac{5n}{2}+7){O}_{2}\to \frac{1}{2}{M}_{2}{O}_{n}+6C{O}_{2}\uparrow +4{H}_{2}O\uparrow +\frac{n}{2}{N}_{2}\uparrow $$

4 $$5{M}_{2}{O}_{n}+n{H}_{2}+nC{H}_{4}\to 10M+3n{H}_{2}O+nC{O}_{2}$$

The TG–DSC–MS results show that the gas phases play an important role in the mechanism behind the sol–gel autocombustion process. Different fuel-oxidant ratios will promote the formation of different gas components, which will significantly affect the composition and structure of the final product. To optimize the fuel–oxidant ratio, reagents with different fuel-oxidant ratios (0.8:1, 0.9:1, 1:1, 1.1:1, and 1.2:1) were dissolved in deionized water to obtain the sols. The dried gels were then activated by combustion at a temperature of 300 °C based on the TG–DSC–MS results.

The XRD patterns obtained for the synthesized CoCrCuNiAl HEAs powders are shown in Fig. 1d. Oxide phase was detected in the combustion product of the gels with a fuel–oxidant ratio of 0.8:1, so the volume of reducing gases created by the fuel was not enough to completely reduce the metal oxides to pure alloys. When the fuel–oxidant ratio was increased to 0.9:1, only weak oxide peaks were detected, and when the fuel-oxidant ratio was increased to 1:1, only peaks corresponding to an FCC structure ((111), (200), and (220)) and a BCC structure ((110), (200) and (211)) could be identified. Further increasing the fuel–oxidant ratio to 1.1:1 and 1.2:1 did not result in an obvious change in the XRD patterns. Combustion product of the gel with fuel-oxidant ratio of 1:1 was annealed at 1,000 °C in a flowing argon atmosphere with a soaking time of 1 h and cooled naturally to the room temperature. A Comparison of the XRD results before and after annealing is shown in Supplementary Figure S1. No oxide peak is detected after annealing, indicating no amorphous oxide in the sol–gel combustion product. In consequence, simple solid solutions rather than complex phases were formed, indicating successful synthesis of HEAs via the sol–gel autocombustion method, and the lowest fuel-oxidant ratio to synthesize pure CoCrCuNiAl HEAs was determined to be a 1:1 ratio. For comparison, a CoCrCuNiAl HEA was also prepared by conventional mechanical alloying (MA). More detailed information on the prepared CoCrCuNiAl HEA via MA is provided in Supplementary Figure S2. As shown in Fig. 1e, both of the XRD patterns of the CoCrCuNiAl HEAs synthesized by MA and sol–gel autocombustion show the same phases, further confirming the successful synthesis of HEAs by the sol–gel autocombustion.

There are two possible routes of the synthesis of CoCrCuNiAl HEAs in the autocombustion process. (i) different metal oxides are reduced to metals by the reducing gases and then form HEAs as the building blocks. (ii) different metal oxides form complex multicomponent oxides, followed by *in situ* reduction to HEAs by the reducing gases. According to thermodynamics, it is impossible to obtain metallic aluminum and chromium in the autocombustion process because Al_2_O_3_ and Cr_2_O_3_ cannot be reduced to metals by any type of the gases formed in the combustion. Therefore, the synthesis of CoCrCuNiAl HEAs is through the second route. Recently, (Co,Cu,Mg,Ni,Zn)O high-entropy oxides (HEOs) were synthesized by pyrolyzing nitrates of the individual metals according to the report of Sarkar *et al*.^25^. Similar with HEAs, HEOs containing five or more metals in equiatomic amounts will be formed because of the high mixing entropy. Hence, the oxide phase in the XRD patterns of the combustion product with fuel–oxidant ratios less than 1:1 is likely to be (Co,Cr,Cu,Ni,Al)O HEO. In the HEOs, the metallic elements are bonded together and ordered in thermodynamics to form the structure of A–B–C–D–E–O (where A, B, C, D, and E denote the metallic elements and O represents oxygen), as shown in Fig. 1f. There is always an intermediate anion separating neighboring cation lattice sites, and no single component metal oxide phases exist in the (Co,Cr,Cu,Ni,Al)O HEOs, so that the reduction of Al^3+^ and Cr^3+^ can be realized. The dried gels with fuel–oxidant ratios of 0.8:1 and 0.9:1 were also activated through combustion in a tube furnace under a flowing hydrogen atmosphere. The XRD patterns of the combustion products are shown in Supplementary Figure S3. No oxide phase was detected in the combustion product, so the external H_2_ can further reduce the oxide phases, validating the above hypothesis.

**Figure 2:**
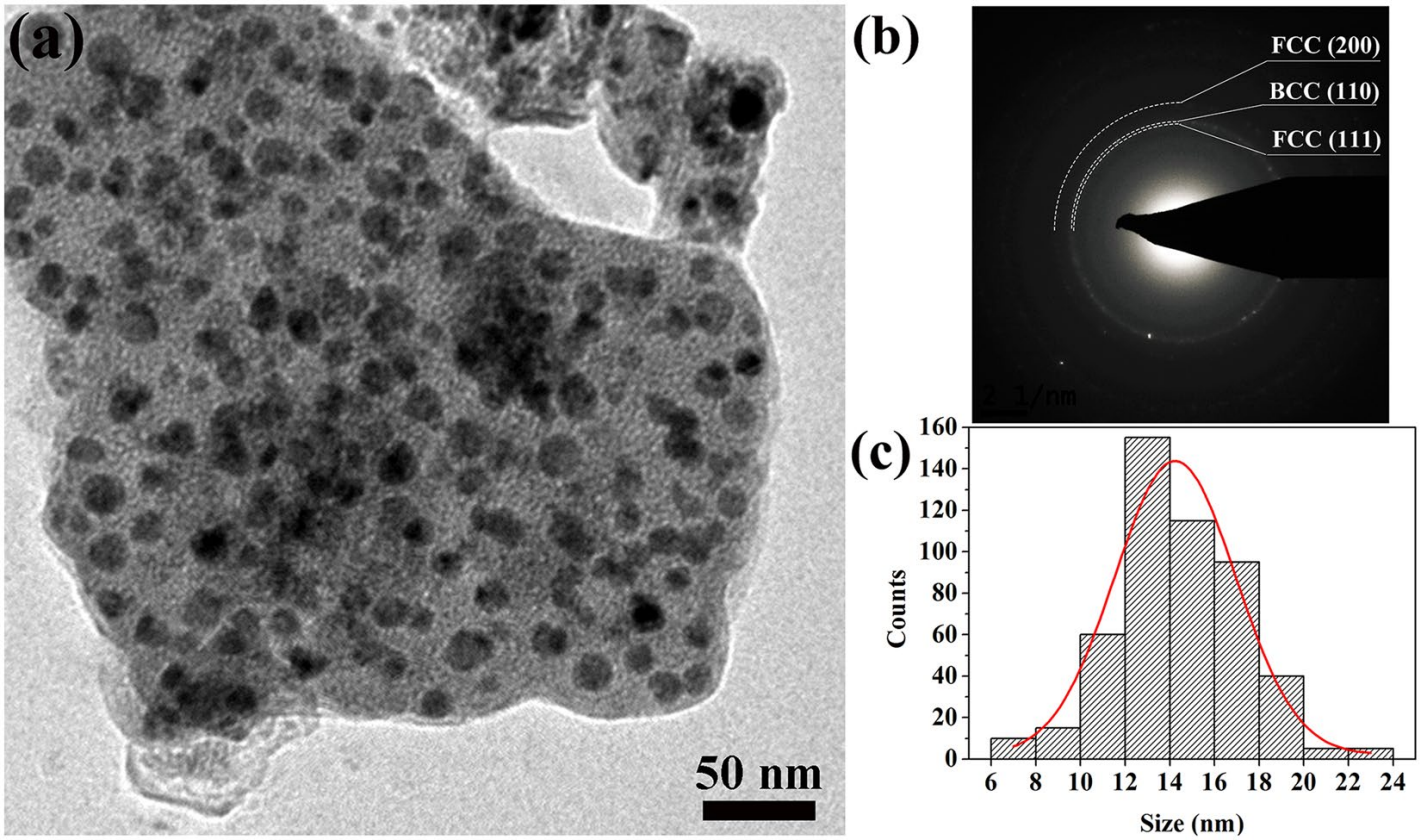
(**a**) TEM image of the prepared CoCrCuNiAl HEA; (**b**) corresponding SAED pattern; (**c**) histogram plot of the particle size distribution.

Transmission electron microscopy (TEM) was used to study the microstructure of the prepared CoCrCuNiAl HEAs. The TEM micrograph revealing the detailed microstructure of the CoCrCuNiAl HEA prepared from a sol with a fuel–oxidant ratio of 1:1 is shown in Fig. 2a, and the TEM images of the combustion products obtained from sols with fuel–oxidant ratios of 0.8:1, 0.9:1, 1.1:1, and 1.2:1 are shown in Supplementary Figure S4, indicating unifromly dispersed HEA nanoparticles. The average grain size of the nanoparticles was calculated to be 14 nm based on a statistical analysis of the size of the particles shown in Fig. 2b. It is worth noting that the grain size of the CoCrCuNiAl HEA synthesized in this study via the sol–gel autocombustion is smaller than the sizes of all HEA nanoparticles so far reported in literature. Furthermore, the nanoparticles show an excellent uniform dispersion. The rings in the SAED pattern reveal that the nanocrystalline CoCrCuNiAl HEA powder prepared via the sol–gel autocombustion consists of a BCC phase and a FCC phase and is in agreement with the XRD results.

**Figure 3:**
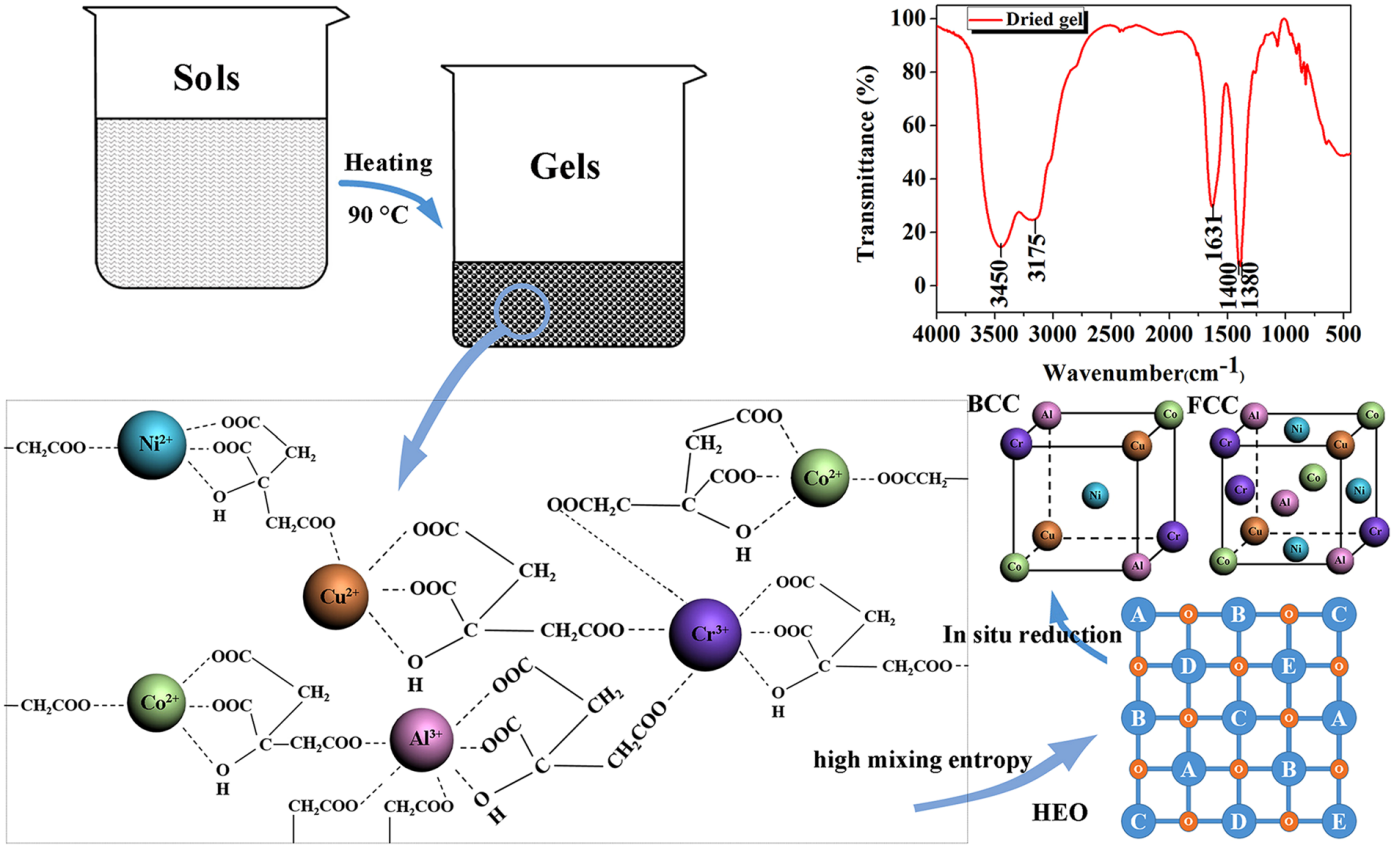
Schematic illustration of the sol–gel combustion process and the FTIR pattern for the dried gel.

Based on the above results, it can be claimed that nanocrystalline CoCrCuNiAl HEA with an average grain size of 14 nm was synthesized via the sol–gel autocombustion. According to the knowledge about HEAs, the different metals should homogeneously share a common lattice to form simple solid solutions. Herein, we suggest that the formation of nanocrystalline HEAs is attibuted to the chelation between citric acid and metal ions. Citric acid has three carboxyl (-COOH) groups and one hydroxyl group (-OH), providing four lone pair electrons to form a tetradentate ligand. However, one metal ion cannot be chelated with more than two of the carboxyl groups, and another carboxyl group can only chelate with other metal ions^25^. The dried gels with a fuel–oxidant ratio of 1:1 were studied by Fourier Transform infrared spectroscopy (FTIR) in the wavelength range from 440 to 4000 cm^−1^, and the resulting spectra are shown in Fig. 3. The band at 1380 cm^−1^ is commonly assigned to the NO^−^ ions, whereas the bands at 1400 and 1631 cm^−1^ are because of the symmetric vibrations, *ν*_*s*_(COO^−^), and antisymmetric stretching vibrations, *ν*_*as*_(COO^−^), of the citrate molecules, respectively. The band at 3175 cm^−1^ is linked to the hydroxyl group (-OH), and the broad bands ∼3500 cm^−1^ can be assigned to water molecules. The distance between the antisymmetric and symmetric stretching vibration peaks, Δ(*ν*_*as*_(COO^−^)-*ν*_*s*_(COO^−^)), was larger than 200 cm^−1^, indicating that some carboxylate groups in the citrate molecules are coordinated to metal ions in a monodentate configuration^26^. Therefore, in the sols, Co^2+^, Cu^2+^, and Ni^2+^ will form tetradentate complexes with an α-hydroxyl group, an α-carboxyl group, and a β-carboxyl group of the citric acid, as well as one of the β-carboxyl groups of another citric acid molecule; Cr^3+^ and Al^3+^ will form hexadentate complexes with an α-hydroxyl group, an α-carboxyl group, and a β-carboxyl group of the citric acid, as well as three β-carboxyl groups of three other citric acid molecules. Because of the chelation of the citric acid and the metal ions, a 3D network will form in the sols, which remains intact during the transformation of the sols into gels, as indicated by the spectra shown in Fig. 3. As a result, the Co^2+^, Cr^3+^, Cu^2+^, Ni^2+^, and Al^3+^ ions will be homogeneously distributed in the dried gels, and no interface will exist between the metal ions. Compared to traditional HEAs synthesis techniques requiring long periods of time for the diffusion of the atoms to form solid solutions, the *in situ* chemical reactions described in Eq. (1) groups (4) only require a short-range diffusion of the atoms to form solid solutions.

**Figure 4:**
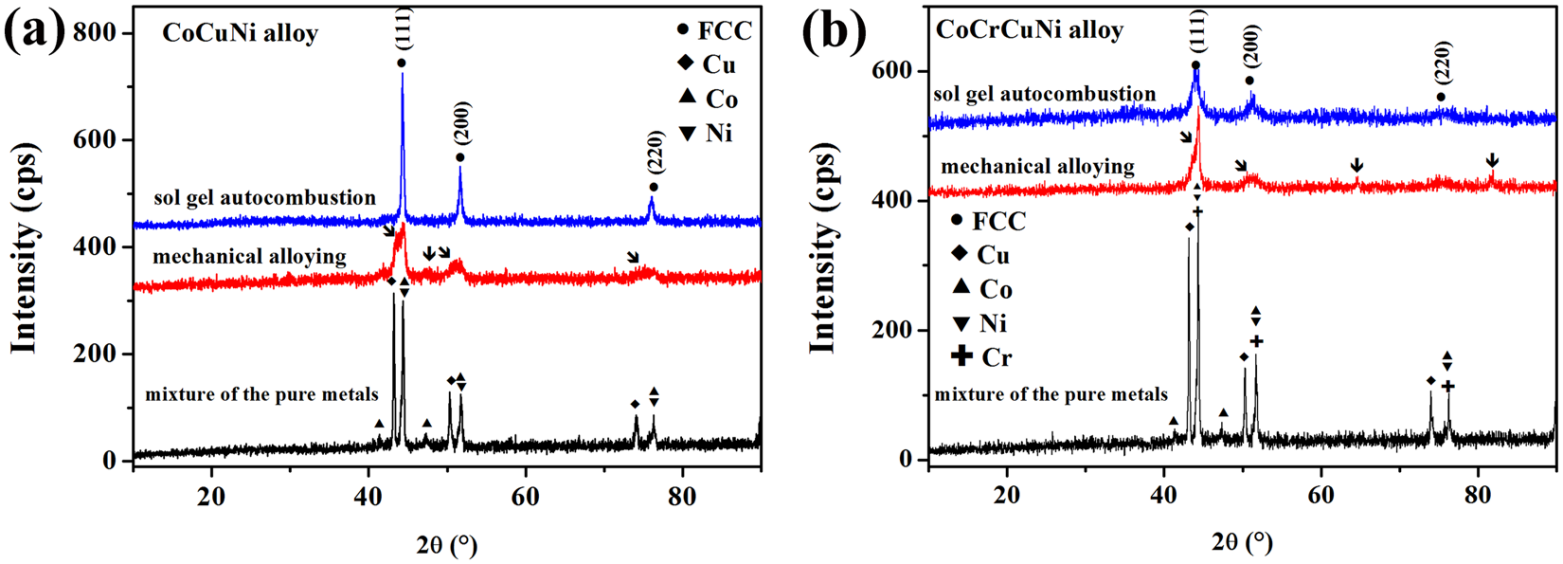
XRD patterns obtained for the equiatomic CoCuNi and CoCrCuNi alloys prepared via sol-gel autocombustion and mechanical alloying, respectively.

To better understand the results, an equiatomic CoCuNi alloy and an equiatomic CoCrCuNi alloy were also synthesized via the sol–gel autocombustion. As shown in Fig. 4, both of these alloys feature an FCC structure. More information on the CoCuNi and CoCrCuNi alloys can be found in Supplementary Figure S5. A comparison of the XRD patterns of equiatomic CoCuNi alloy and equiatomic CoCrCuNi alloy synthesized by the sol–gel autocombustion and MA are shown in Fig. 4. Some additional peaks still can be detected after mechanical alloying for 60 h; however, there are no additional peaks, and the background is low and flat, and peak widths are narrow in 2θ space in the XRD patterns of equiatomic CoCuNi alloy and CoCrCuNi alloy synthesized by the sol–gel autocombustion, indicating the successful synthesis of ternary and quaternary equiatomic solid solution alloys. According to Boltzmann’s hypothesis regarding the relationship between the entropy of a system and the system complexity, for a random solid solution, the configurational entropy of mixing is represented by the following Eq.

5 $${\rm{\Delta }}{S}_{mix}=-R\sum _{i}{c}_{i}ln{c}_{i}$$

where *R* is the gas constant (8.314 J/K mol) and ${c}_{i}$ is the molar content of the *i* ^th^ component^27^. Thus, for an equiatomic alloy with *n* components, the configurational entropy of mixing is:

6 $${\rm{\Delta }}{S}_{mix}=Rlnn$$

For an equiatomic alloy with 3, 4, and 5 principal components, Δ*S*_*mix*_ is 1.10 *R*, 1.39 *R*, and 1.61 *R*, respectively. In fact, complex phases or intermetallics easily form in alloy systems with multiple principal components according to traditional metallurgical knowledge^28^, and simple solid solutions will be formed when the alloys contain more than five principal elements because of the high mixing entropy (>1.61 *R*) according to the knowledge about HEAs. However, CoCuNi (ternary, ${\rm{\Delta }}{S}_{mix}=1.10R$) and CoCrCuNi (quaternary, ${\rm{\Delta }}{S}_{mix}=1.39R$) equiatomic solid solution alloys without a high mixing entropy were synthesized in this study. Therefore, according to the above discussion, some equiatomic solid solution alloys can be synthesized eluding the restriction of a high mixing entropy, which further indicates the superiority of the sol–gel autocombustion technique for the preparation of solid solution HEAs.

**Figure 5:**
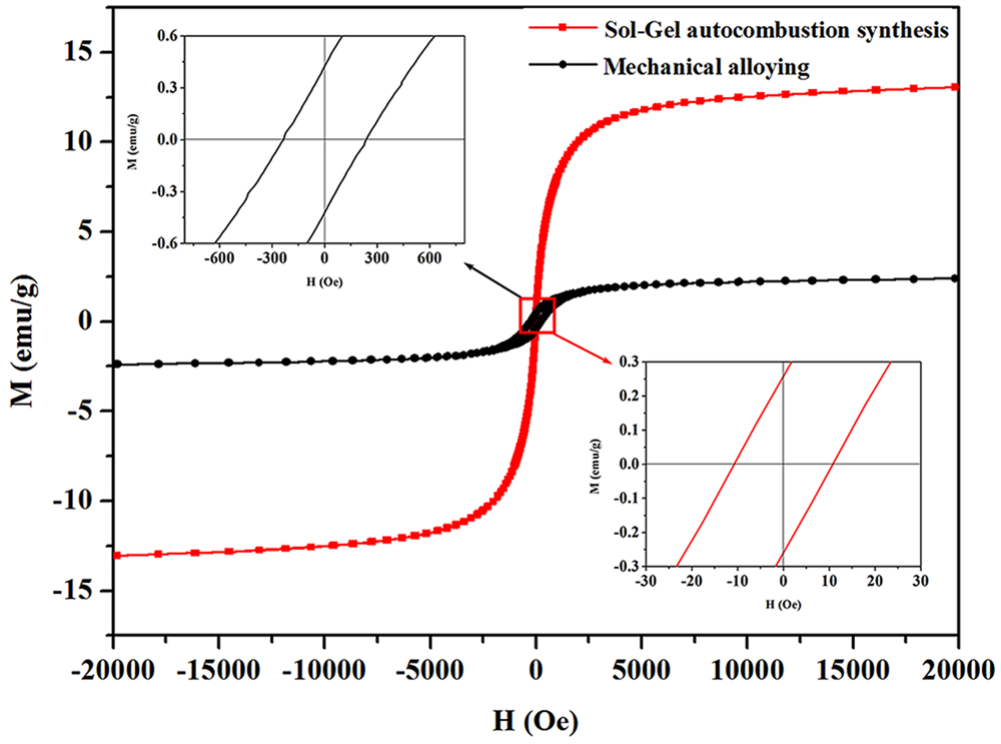
Comparison of the magnetic hysteresis curves obtained for the CoCrCuNiAl HEAs prepared via the sol–gel autocombustion and mechanical alloying, respectively.

The magnetic properties of the CoCrCuNiAl HEAs synthesized via the sol–gel autocombustion and mechanical alloying were measured at room temperature. The obtained magnetic hysteresis loops are shown in Fig. 5. The HEA powder prepared via MA showed a ferromagnetic behavior, and the saturated magnetizations (*M*_*S*_), remanence ratio (*M*_*R*_/*M*_*S*_) and coercivity force (*H*_*C*_) were measured to be 2.521 emu/g, 17.3%, and 238.867 Oe, respectively. However, compared to the magnetic properties of the CoCrCuNiAl HEA synthesized via MA, the corresponding HEA synthesized via the sol–gel autocombustion showed a distinctive soft magnetic behavior and higher saturated magnetizations (13.372 emu/g), a lower remanence ratio (1.93%), and a lower coercivity force (10.528 Oe). Furthermore, the HEA powder synthesized via the sol–gel autocombustion showed a behavior similar to a superparamagnetic behavior because of the low remanence ratio. In general, magnetic nanoparticles with diameters <10 nm exhibited a superparamagnetic behavior^29^. The CoCrCuNiAl HEA synthesized via the sol–gel autocombustion had an average grain size of 14 nm, and can easily lead to the observed magnetic behavior similar to a superparamagnetic behavior. This makes HEAs prepared via the sol–gel autocombustion promising candidates for future applications in some special conditions.

## Conclusions

In summary, CoCrCuNiAl HEAs were successfully synthesized via the sol–gel autocombustion. The optimal fuel–oxidant ratio for the synthesis of CoCrCuNiAl HEAs using nitrate ions as the oxidants and citric acid as the fuel was determined to be 1:1. Solid solutions with an FCC and BCC structure and an average grain size of 14 nm were obtained after the combustion process, and the HEA nanoparticles showed an excellent uniform dispersion. The synthesis of the nanocrystalline HEAs was associated with the chelation between citric acid and metal ions and the *in situ* chemical reactions. The nanopowders exhibited a distinctive magnetic behavior similar to a superparamagnetic behavior, demonstrating the great application potential of HEAs synthesized via the sol–gel autocombustion.

## Methods

### Preparation of the HEAs by sol–gel autocombustion

Analytical grade Co(NO_3_)_2_·6H_2_O, Cr(NO_3_)_3_·9H_2_O, Cu(NO_3_)_2_·3H_2_O, Ni(NO_3_)_2_·6H_2_O, Al(NO_3_)_3_·9H_2_O, citric acid, ethanol, and ammonia (all procured from Sinopharm, China) were used as the starting materials in this study. The metal nitrates were first dissolved in distilled water at an equiatomic ratio and stirred thoroughly at room temperature for 1 h. Then, citric acid was added to the solution as coordinating agent with the molar ratio of citric acid to the total amount of metal ions selected to 0.8:1, 0.9:1, 1:1, 1.1:1, or 1.2:1. After homogenizing the solution through magnetic stirring, the pH value was adjusted to 7 by adding ammonia, and finally the sols were annealed at 90 °C for 48 h to form the dried gels. The dried gels were activated by combustion at 300 °C in a tube furnace under a protective argon atmosphere or under a flowing hydrogen atmosphere.

### Preparation of the HEAs by mechanical alloying

For comparison, CoCuNi, CoCrCuNi, and CoCrCuNiAl HEAs were also prepared via mechanical alloying (MA). Co, Cr, Cu, Ni, and Al metal powders were used as the starting materials in this case. The powders were milled in a high-energy planetary ball mill (QM-BP, Nanjing Nan-Da Instrument Plant) at 250 rev/min with a ball-to-powder weight ratio of 15:1 for 60 h, and n-heptane was used as the process-controlling agent.

### Characterization of alloys

X-ray diffraction (XRD, Rigaku Ultima III) measurements were performed using Cu Kα radiation in the 2θ range from 10 to 90° at a scanning rate of 4°/min to analyze the crystal structure of the obtained HEAs powders. A thermogravimetric analysis combined with differential scanning calorimetry and dynamic mass spectrometry (TG–DSC–MS, Netzsch STA449F3, Germany) was used to perform an *in situ* thermogravimetric and gas-phase analysis of the reactive gels under a high-purity argon atmosphere using a heating rate of 10 K/min. Transmission electron microscopy (TEM, Philips M12) was used in conjunction with selected area electron diffraction (SAED) to analyze the microstructure of the powders. The gels were evaluated by Fourier Transform infrared spectroscopy (FTIR, Thermo Scientific Nicolet 6700) from 4000 to 400 cm^−1^, at a resolution of 4 cm^−1^ with 32 scans. The magnetic properties of the HEAs at room temperature were measured using a Physical Property Measurement System (PPMS, Quantum Design PPMS-9T), and the magnetic field was varied from −20000 to 20000 Oe.

## Electronic supplementary material

Supporting Information
