# Supplementary material for: Sol-gel Autocombustion Synthesis of Nanocrystalline High-entropy Alloys
Source: Sci Rep. 2017 Jun 13;7:3421. doi: 10.1038/s41598-017-03644-6 (PMC5469749; doi:10.1038/s41598-017-03644-6)
Supplement: Supplementary file 1 — Supporting Information [file 41598_2017_3644_MOESM1_ESM.pdf]

Supporting Information

Sol-gel Autocombustion Synthesis of Nanocrystalline High-entropy Alloys

Bo Niu, Fan Zhang*, Hang Ping, Na Li, Jieyang Zhou, Liwen Lei, Jingjing Xie, Jinyong Zhang, Weimin Wang and Zhengyi Fu*

State Key Laboratory of Advanced Technology for Materials Synthesis and Processing, Wuhan University of Technology, Wuhan 430070, China

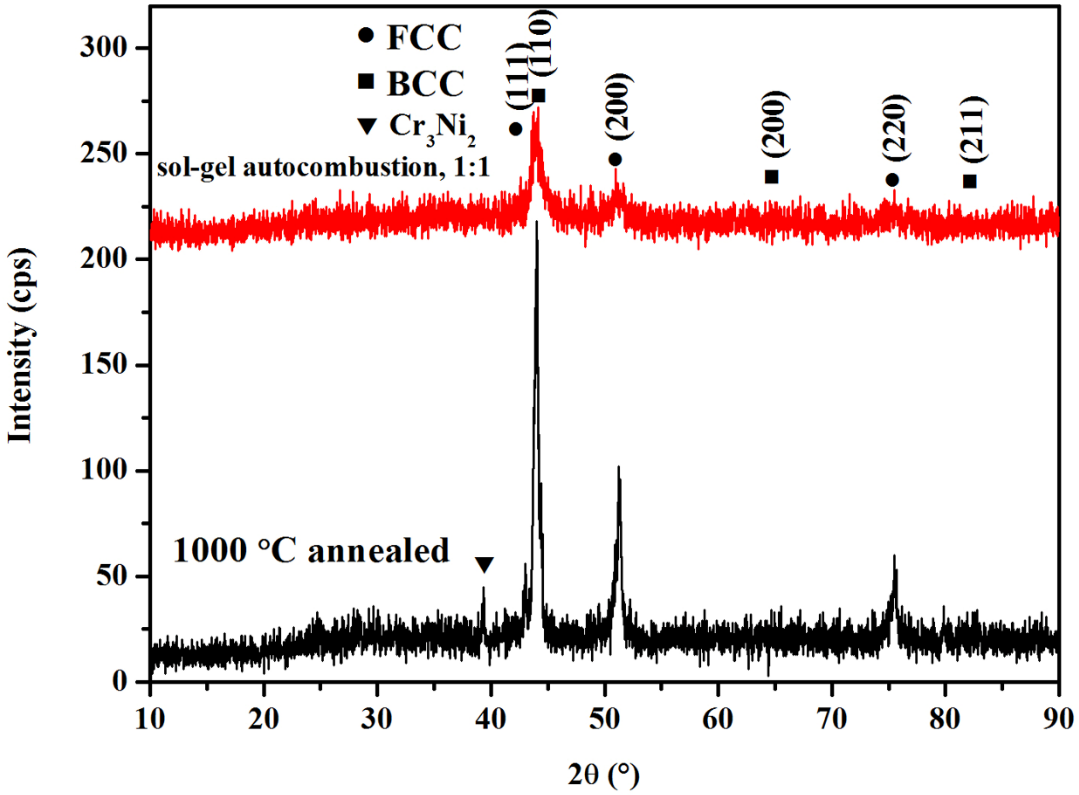

Supplementary Figure S1. XRD patterns of the CoCrCuNiAl HEA with a fuel–oxidant ratio of 1:1 and the CoCrCuNiAl HEA powder annealed at 1000 °C. The synthesized CoCrCuNiAl HEA is composed of FCC and BCC solid solutions, and the annealed HEA shows the same structure except for an increase in the diffraction peaks. Cr_3Ni_2 phase is detected after annealing at 1,000 °C because of the decomposition of the HEAs at the high temperature. No oxides is detected after annealing at 1000 °C, indicating that there is no amorphous oxides in the sol–gel combustion product.

* Corresponding author: zhfan@whut.edu.cn; zyfu@whut.edu.cn

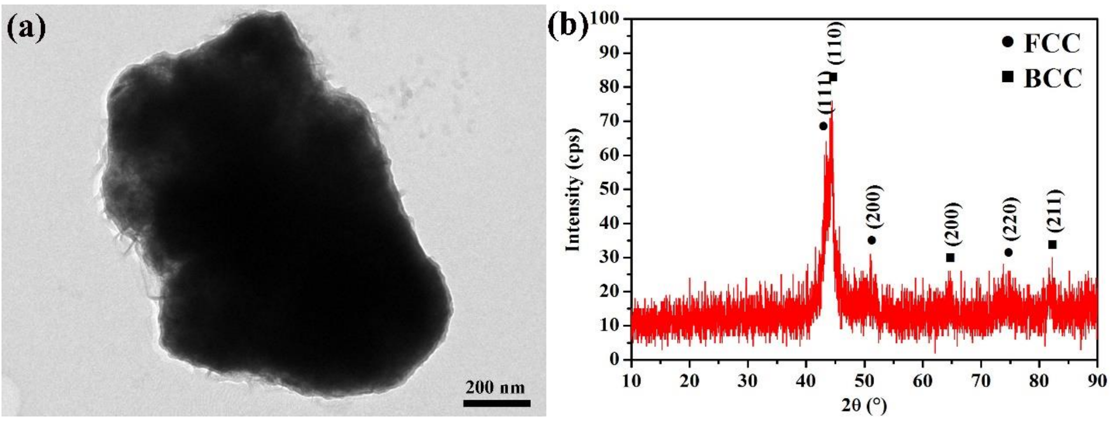

Supplementary Figure S2. Additional information on the CoCrCuNiAl HEA prepared via mechanical alloying: a) TEM image, b) XRD pattern. The ball-milled alloy powder consists of simple solid solutions of the FCC and BCC structure with a particle size of about 3 μm.

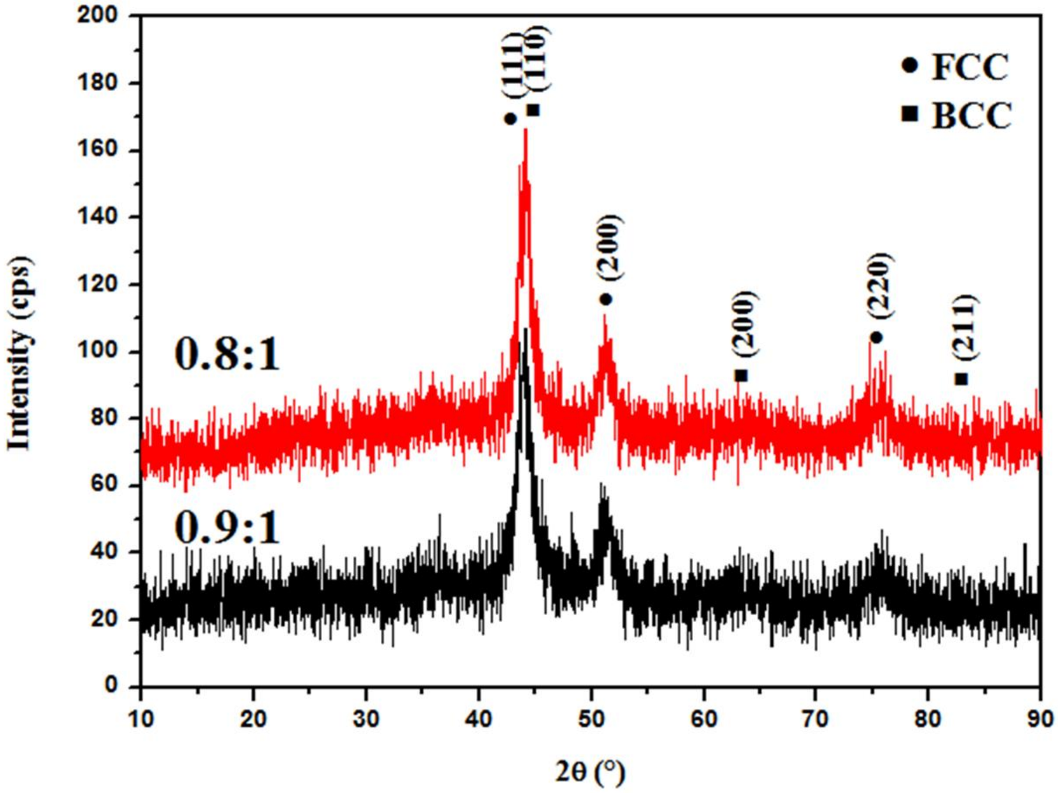

Supplementary Figure S3. XRD patterns of the combustion product of the gels with fuel-oxidant ratios of 0.8:1 and 0.9:1 in the flowing hydrogen atmosphere.

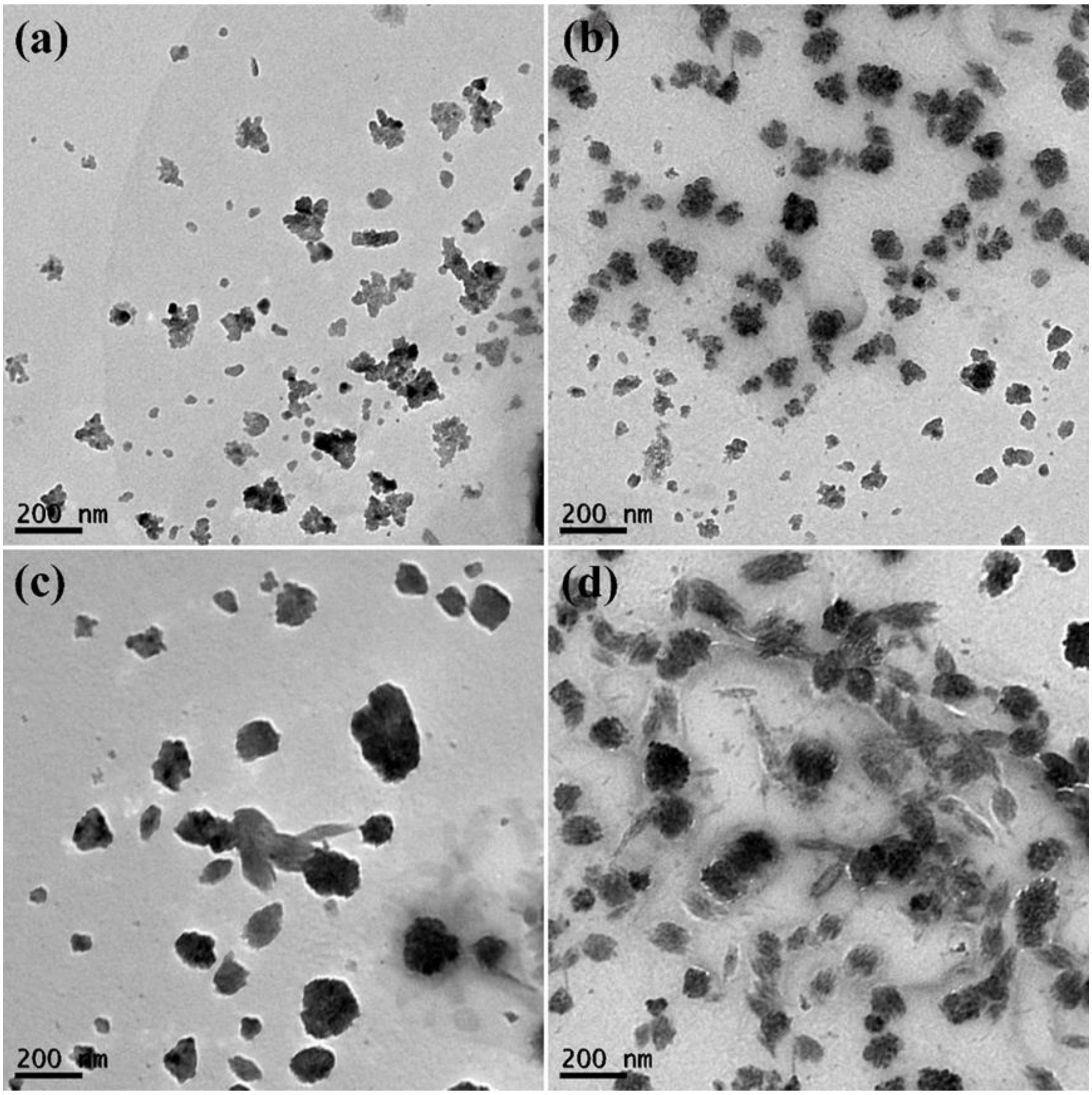

Supplementary Figure S4. TEM images showing the combustion products prepared from sols with a fuel-oxidant ratio of a) 0.8:1, b) 0.9:1, c) 1.1:1 and d) 1:2:1, respectively.

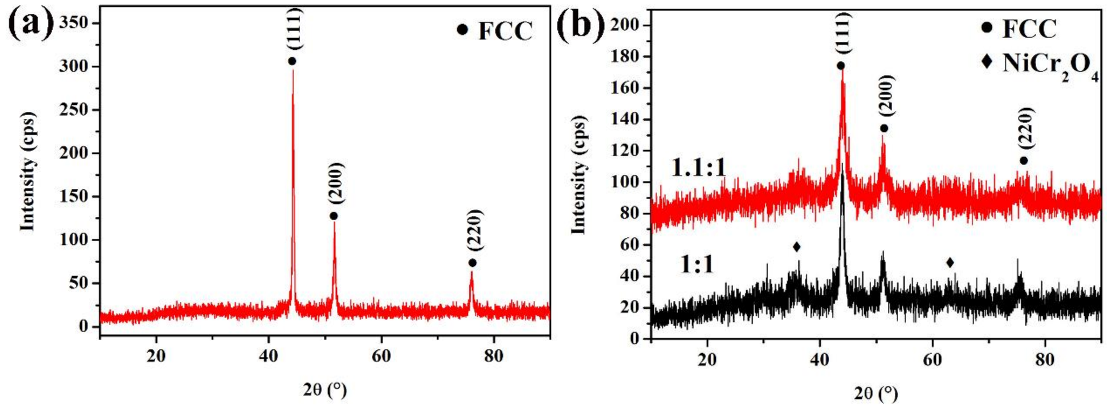

Supplementary Figure S5. XRD results obtained for the CoCuNi and CoCrCuNI alloys with prepared from sols with different fuel-oxidant ratios. The CoCuNi and CoCrCuNI alloys prepared from the sols with the lowest fuel-oxidant ratio were selected according to the optimal fuel-oxidant ratio determined for the CoCrCuNiAl HEA.
